# Lubricin/Proteoglycan 4 binds to and regulates the activity of Toll-Like Receptors *In Vitro*

**DOI:** 10.1038/srep18910

**Published:** 2016-01-11

**Authors:** S.M. Iqbal, C. Leonard, S. C. Regmi, D. De Rantere, P. Tailor, G. Ren, H. Ishida, CY. Hsu, S. Abubacker, D. SJ. Pang, P. T. Salo, H.J. Vogel, D.A. Hart, C.C. Waterhouse, G.D Jay, T.A. Schmidt, R.J. Krawetz

**Affiliations:** 1 McCaig Institute, Cumming School of Medicine, University of Calgary, Calgary, Alberta, Canada; 2 Faculty of Kinesiology, University of Calgary, Calgary, Alberta, Canada; 3 Faculty of Veterinary Medicine, University of Calgary, Calgary, Alberta, Canada; 4 Faculty of Science, University of Calgary, Calgary, Alberta, Canada; 5 Snyder Institute, Cummings School of Medicine, University of Calgary, Calgary, Alberta, Canada; 6 Faculty of Medicine, Brown University, Providence, Rhode Island, United States; 7 Schulich School of Engineering, University of Calgary, Calgary, Alberta, Canada

## Abstract

Proteoglycan 4 (PRG4/lubricin) is secreted by cells that reside in articular cartilage and line the synovial joint. Lubricin may play a role in modulating inflammatory responses through interaction with CD44. This led us to examine if lubricin could be playing a larger role in the modulation of inflammation/immunity through interaction with Toll-like receptors (TLRs). Human Embryonic Kidney (HEK) cells overexpressing TLRs 2, 4 or 5 and surface plasmon resonance were employed to determine if full length recombinant human lubricin was able to bind to and activate TLRs. Primary human synovial fibroblasts were also examined using flow cytometry and Luminex multiplex ELISA. A rat destabilization model of osteoarthritis (OA) was used to determine if lubricin injections were able to regulate pain and/or inflammation *in vivo*. Lubricin can bind to and regulate the activity of TLRs, leading to downstream changes in inflammatory signalling independent of HA. We confirmed these findings *in vivo* through intra-articular injections of lubricin in a rat OA model where the inhibition of systemic inflammatory signaling and reduction in pain were observed. Lubricin plays an important role in regulating the inflammatory environment under both homeostatic and tissue injury states.

Osteoarthritis (OA) affects all tissues in the synovial joint, however, the hallmark of the disease is the loss of articular cartilage. Within the joint, articular cartilage bears load and slides relative to an opposing tissue surface, forming a biointerface with remarkable low-friction and low-wear properties. These properties are facilitated by multiple modes of lubrication and the surrounding synovial fluid (SF)^1^. SF contains secreted products of cells that line the joint cavity, some of which provide lubricating function to articular cartilage. Furthermore, there is evidence that suggests failure in lubrication mechanisms may contribute to the roughening and erosion of cartilage often observed in aging and OA^2^,^3^,^4^.

Proteoglycan 4 (PRG4), a mucin-like glycoprotein also known as lubricin was originally discovered in SF, is present at the surface of articular cartilage where it contributes to the maintenance and integrity of the joint^5^. Lack of lubricin expression in humans and animal models has no adverse effect on musculoskeletal development or joint formation *in utero*, yet results in premature joint failure^6^. Lubricin (together with hyaluronan (HA) a repeating disaccharide also present in SF and at the surface of cartilage), functions as a dose-dependent boundary lubricant of cartilage. These molecules act synergistically^7^,^8^ to reduce friction to levels near that of native SF^9^,^10^.

Human lubricin contains two mucin-like domains. The first mucin domain contains multiple copies of the peptide motif KEPAPTTT, with threonine residues critical for O-linked GalNAc-Gal-NeuAc sidechains. In arthritis, branching of these O-linked lubricin glycosylations have been observed in patients with OA and rheumatoid arthritis (RA)^11^,^12^. The second mucin-like domain is also threonine rich but does not contain the KEPAPTTT motif. A hemopexin-like (PEX) domain at the carboxyl-terminus of the protein is hypothesized to be necessary for extracellular matrix binding^12^.

Decreased lubricin concentration in SF following an injury is associated with increased cartilage damage, and may play a role in the subsequent degeneration leading to OA^13^. In patients with acute knee injuries, cartilage damage has been associated with decreased boundary lubricating ability of SF^14^. Decreased lubricin concentrations have been reported both in SF of knee injury patients^13^ and in some OA patients^14^. Lack of lubricin has also been shown to contribute to chondrocyte death in the absence of other joint pathologies^15^. While the full extent of lubricin’s molecular interactions in the joint remain to be elucidated, local administration of lubricin has been shown to be therapeutically effective in preventing cartilage degeneration in rat models of OA^16^,^17^,^18^.

Interestingly, lubricin expression shows an inverse relationship to inflammation^19^,^20^. After joint injury, inflammatory cytokines such as TNF-α and IL-1β are increased, while lubricin concentrations are decreased, and do not return to normal levels until inflammation has subsided^19^. This has been further explored *in vitro* and *ex vivo* in chondrocyte cell culture and cartilage explant systems, and it appears that TNF-α and IL-1β treatment can down-regulate the expression and secretion of lubricin at the cellular level^20^. While this result has been observed in independent studies, the mechanism by which these factors regulate lubricin remains unknown. Additionally, it has been observed that as lubricin levels decrease, inflammation increases^19^,^21^,^22^, suggesting that lubricin may play a role in inhibiting inflammation within the joint. However, evidence supporting this hypothesis remains indirect and a mechanism to explain the relationship between lubricin and inflammation remains to be identified. There is an expanding body of research exploring the role of HA in various inflammatory processes showing that HA regulates the activation of TLRs^23^,^24^,^25^. Given the synergistic relationship between lubricin and HA in terms of lubrication^26^,^27^, lubricin may also have the ability to regulate inflammatory pathways either alone or in concert with HA.

TLRs are an important class of pattern recognition receptors expressed predominantly by cells involved in the innate immune system. They recognize, and are activated by, structurally conserved molecules in bacteria, viruses and certain endogenous ligands reviewed in^28^,^29^. The domains TLRs recognize are known as pathogen-associated microbial patterns (PAMPs), which are expressed by pathogens, or danger-associated molecular patterns (DAMPs/Alarmins) that are released from necrotic or dying cells^30^. PAMPs include bacterial components such as lipopolysaccharide (LPS), as well as flagellin (FLA), bacterial DNA and viral double-stranded RNA^31^. DAMPs include intracellular proteins and protein fragments from the extracellular matrix (ECM)^31^. Stimulation of TLRs by either PAMP’s or DAMP’s initiates signalling cascades leading to the activation of NF-κB and other pathways. These signalling pathways can result in a variety of distinct cellular responses including the production of pro and/or anti-inflammatory cytokines^32^.

In this study, we present data demonstrating that lubricin can regulate the inflammatory response through TLRs *in vitro*, and that intra-articular injection of lubricin into a rodent model of OA regulates the inflammatory response and pain. These findings suggest that lubricin may play a number of pivotal roles in the joint to maintain homeostasis and control inflammation, beyond a purely mechanical/lubricating role.

## Methods

### Ethics Statement

Informed consent to participate was obtained by written agreement. The study protocol was approved by the University of Calgary Research Ethics Board. All methods were carried out in accordance with the approved guidelines

Animal studies were carried out in accordance with the recommendations in the Canadian Council on Animal Care Guidelines. Animal protocols and surgical procedures in this study were approved by the University of Calgary Health Sciences Animal Care Committee.

### Patient Inclusion and Exclusion Criteria

Normal Group (n = 4): Criteria for control cadaveric donations were an age of 40 years or older, no history of arthritis, joint injury or surgery (including visual inspection of the cartilage surfaces during recovery), no prescription anti-inflammatory medications, no co-morbidities (such as diabetes/cancer), and availability within 4 hrs of death.

OA Group (n = 4): Criteria were an age of 40 years or older, OA diagnosed based on the American College of Rheumatology criteria with X-ray documentation, and no evidence of autoimmune disease or RA. Synovial biopsies from the medial compartment were collected during routine arthroscopy. Only patients with an Outerbridge score of 3 or greater were selected for this study.

### Clinical grade recombinant lubricin preparation

Full length recombinant human lubricin protein (rhPRG4) was derived from Chinese Hamster Ovary cells (Lubris, LLC, Framingham, MA). Briefly, the gene encoding the full length 1404 amino acid human PRG4 was inserted into plasmid vectors, commercially available at Selexis SA (Switzerland), and subsequently the protein was subjected to ultrafiltration/diafiltration and a 3-step chromatographic purification process^33^.

### Surface Plasmon Resonance (SPR)

Binding of lubricin to the TLR members of interest was assessed using a Biacore X100 SPR instrument (GE Healthcare, Pittsburg PA). Human TLR-2, -4 or -5 (R&D Systems, Minneapolis, MN) were immobilized onto the flow cell 2 of CM5 sensor chip (GE Healthcare, Little Chalfont, United Kingdom) using standard amine-coupling chemistry, resulting in 300-500 response units (RU). The reference cell (flow cell 1) was prepared by activation and deactivation. The binding assay was performed in PBS running buffer supplemented with 0.01% (v/v) Tween 20. Lubricin solution was buffer exchanged to running buffer and at least 5 concentrations in the range of 0.576–420 μg/mL were injected at a flow rate of 30 μL/min with a contact time of 1 min at 25 °C. The bound lubricin was removed from the chip surface by injecting 1 M NaCl after monitoring dissociation for 1.5 min.

### Transgenic TLR cell assays

TLR Null, TLR Null-2, TLR-2, -4, and -5 cell lines (Invivogen, San Diego, CA) were exposed to either positive controls for the TLRs (Heat-killed Listeria Monocytogenes:HKLM for TLR-2 (10^8^ cells/ml), LPS for TLR-4 (100 ng/ml), and FLA for TLR-5 (100 ng/ml) in separate experiments; Antagonists in competitive binding experiments included recombinant human lubricin (90 μg/ml); or HA (100 μg/ml, MW 5, 20, 132, or 780 kDa; Lifecore Biomedical, Chaska, MN). The cells and ligands were then plated and incubated at 37 °C, 5% CO2 over a 22-24 hour period in HEK Blue media (Invivogen) then measured at 630 nm.

### Cytokine/Chemokine analysis

Cell supernatant was collected and processed using the Luminex (Madison, WI) platform and the Millipore (Billerica, MA) human cytokine array (EGF, Eotaxin-1, FGF-2, Flt-3L, Fractalkine, G-CSF, GM-CSF, GRO(pan), IFNα2, IFNγ, IL-1α, IL-1β, IL-1rα, IL-2, IL-3, IL-4, IL-5, IL-6, IL-7, IL-8, IL-9, IL-10, IL-12 (p40), IL-12 (p70), IL-13, IL-15, IL-17A, IP-10, MCP-1, MCP-3, MDC, MIP-1α, MIP-1β, PDGF-AA, PDGF-AB/BB, RANTES, sCD40L, TGFα, TNFα, TNFβ, VEGF-A). Samples were stored at –80 °C and analyzed together to minimize inter-experimental variation. All cytokines present were quantified in pg/mL, and analyzed in duplicate alongside a reference sample and compared to an internal standard curve.

### Destabilization of the Medial Meniscus (DMM) injury model

Standardized joint injuries (n = 9) were created while each rat was anesthetized using isoflurane delivered in oxygen. A medial para-patellar arthrotomy was performed under a dissection microscope. The medial margin of the quadriceps was separated from the muscles of the medial compartment and the patella was dislocated laterally. The fat pad over the cranial horn of the medial meniscus was retracted. Sectioning of the medial meniscotibial ligament leads to a destabilization of the medial meniscus. Control groups did not undergo surgery (n = 9). The joint capsule was closed with a silk suture. The skin was closed by the application of tissue adhesive.

### Lubricin administration

Lubricin was administered intra-articularly under isoflurane anesthesia using a custom rat knee injection apparatus one week after DMM surgery using a dose of 200 ug/kg in 10 ul of sterile saline. Control animals received an equal volume of sterile saline.

### Histology and OA grading

Animals were sacrificed three weeks after lubricin injection (four weeks after DMM surgery) and intact knee joints were dissected and fixed in 4% normal buffered formalin (Sigma, St. Louis, MO). Samples were decalcified and embedded in paraffin (VWR, Radnor, PA). Ten μm thick, longitudinal serial sections were stained with Safranin O (Fisher, Waltham, MA) to visualize proteoglycans. Whole joint sections, medial, lateral and the ACL/PCL insertion sites were graded for signs of OA according to the OARSI Guidelines for rat knee joints^34^. Sections were deparaffinized in CitraSolv (Fisher Scientific; Fairlawn, NJ) and rehydrated through a series of graded ethanol to distilled water steps. Antigen retrieval (10 mM sodium citrate, pH 6.0, Fisher Scientific) and blocking (1:500 dilution; 100 μL rat serum:50 mL TRIS-buffered saline, 0.1% Tween 20 (TBST) for 1 hr), steps were performed prior to going through sequential wash (TBST) and primary antibody application steps. A primary antibody for NFkB P65 antibody (Cell Signaling Technology), or lubricin^12^ bound to Alexa 488 (Molecular Probes) and the nucleic acid stain DAPI (Sigma) were applied to sections. After antibody staining, sections were mounted using FluorSave reagent (Calbiochem) and coverslipped.

### Rat Grimace Scale (RGS)

Application of the RGS was based on scoring randomised, blinded images of individual rats^35^. Briefly, each rat was video-recorded for 15 minutes at each time point in a plexiglass video chamber (W 14 cm× L 26.5 cm× H 20.5 cm). Recorded video was reviewed by a trained observer (blinded to treatment and time point) and an image captured every 3 minutes. No images were collected during the first minute of recording to allow the rat to acclimate. Image selection criteria were: absence of movement artifact, a clear view of all relevant facial features (nose, cheek, eyes, ears) and absence of directed behaviours (grooming, rearing, sleeping). Generation of a score with the RGS requires assessment of four “action units”: orbital tightening, nose/cheek flattening, ear changes, and whisker change. Each action unit was assigned a score of 0, 1 or 2, and the four scores averaged to generate a single RGS score for each image. A score of “0” reflects an absent action unit, a score of “1” indicates the moderate appearance of an action unit, and a score of “2” indicates the obvious appearance of an action unit (associated with a painful state).

### Synovial Fibroblast isolation

Human synovial membrane fibroblasts were obtained from fresh synovial tissue biopsies (~3 mm^3^). The biopsies were minced and placed into DMEM:F12 medium containing 10% fetal bovine serum, 1% non-essential amino acids, 1% pen-strep and 0.1% beta-mercaptoethanol (all Life Technologies, Carlsbad, CA). At 7–14 days after initial seeding (approx. passage 3), the cells were processed for magnetic purification using the Human Lineage Cell Depletion Cocktail (BD Canada, Ontario, Canada) that removes CD3, CD14, CD16, CD19, CD41a, CD56 and Glycophorin A positive cells.

### Flow Cytometry

Normal, OA and HEK cells were dissociated and resuspended in 500 μl of 90% MeOH (Sigma) and left for 5–10 minutes at room temperature. The cells were then centrifuged, the liquid was removed and 500 μl of 0.1% Tween 20 (Sigma) was added to permeabilize the cells for 20 minutes at room temperature. The cells were centrifuged again, the liquid was removed, and 50 μl of Tween buffer and 0.5 μg of respective TLR antibody (R&D Systems) was added to each tube and incubated in the dark for 30–45 minutes at room temperature. The cells were then washed three times with FACs buffer then resuspended in FACs buffer and then measured using the Attune Flow Cytometer (Life Technologies). The results were analyzed using FlowJo software (Ashland, OR).

### Transfection

For gene knockdown, we employed the shRNA plasmids psiRNA-TLR2, psiRNA-TLR4, psiRNA-TLR5 from Invivogen (San Diego, CA). Plasmid DNAs were purified from bacterial cultures using the PureLink® HiPure Plasmid Midiprep Kit (Life Technologies Inc.; Burlington, ON) as described in the manufacturer’s handbook. Cells were transfected using the TransIT-2020 transfection reagent from Mirus Bio LLC (Madison WI) according to manufacturer’s protocol. Briefly, plasmid DNA were diluted in Opti-MEM® I Reduced Serum Media at a final concentration of 0.01 ug/ml. Then TransIT-2020 reagent was added to the diluted DNA in drop-wise fashion at a DNA-to-reagent (w/v) ratio of 1:1.5 and incubated at room temperature for 15 min; the prepared transfection/DNA mixture is then diluted with cell culture media to a final DNA concentration of 1 ug/ml and added directly to cells that have reached a confleuncy of ~80–90%. After 24 h incubation at 37 °C, Zeocin (Invivogen) was added to the culture media to select for transfected cells for an additional 48 h before processing for subsequent analysis.

### Statistics

One-way ANOVAs were conducted on the absorbance assays in order to determine significance.

P-values were obtained via post-hoc Bonferroni test to determine the significance values between the conditions and their negative controls at the 24 hour time point. For the cytokine analysis, two-way ANOVAs were conducted to determine significance between conditions and their negative controls. The p-values were obtained using post-hoc Bonferroni test. Due to the large number of comparisons, the p-values were corrected for using the Benjamini-Hochberg method. In this method, all the p-values are ordered smallest to largest and the smallest p-value receives a rank (*i*) of 1, the second largest receives an *i* = 2, and so on. Each p-value is then compared to its (*i/m*)*q* value, where *i* is the rank, *m* is the total number of comparisons made, and *q* is the false discovery rate.

The new threshold for significance is set at the highest p-value that is lower than its (*i/m*)*q (P < (i/m)q).* The false discovery rate (*q*) was set at 0.1. All error bars presented are SEM.

RGS scores were compared between treatment groups with a Mann-Whitney test and data displayed as Tukey box and whisker plots.

## Results

### Lubricin Regulates NF-ĸB Translocation through TLR Receptors

**Figure 1:**
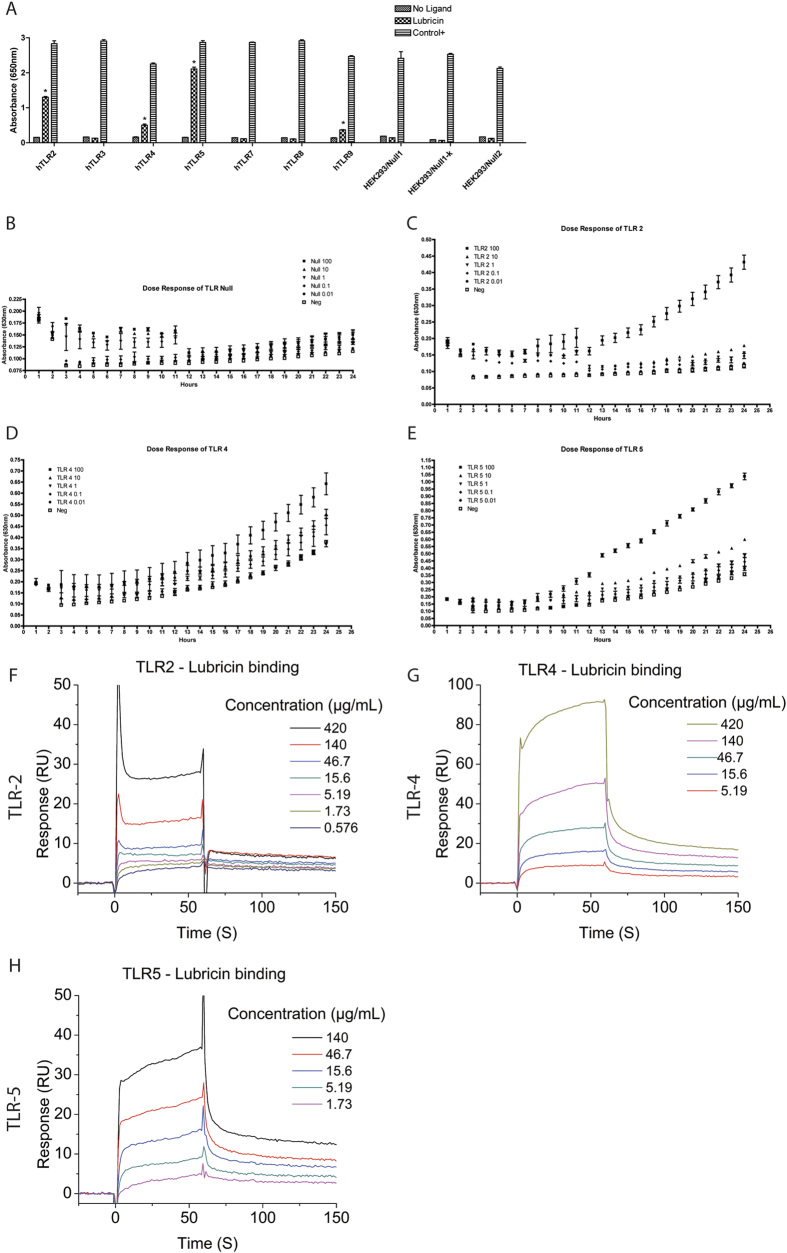
Lubricin binds to and regulates TLRs in a dose dependent manner. Human recombinant lubricin was screened as a ligand for multiple TLRs (**A**) and was found to trigger the activation of the NF-ĸB pathway downstream of TLR -2, -4, -5 and -9. The activation of TLR 2, 4 and 5 was found to occur in a dose dependent manner with 90–100 ug/mL of lubricin triggering robust NF-ĸB activation (**B–E**). Direct binding was assessed using SPR and again lubricin was observed to bind TLR-2 (**F**), -4 (**G**) and -5 (**H**) in a dose dependent manner.

**Figure 2:**
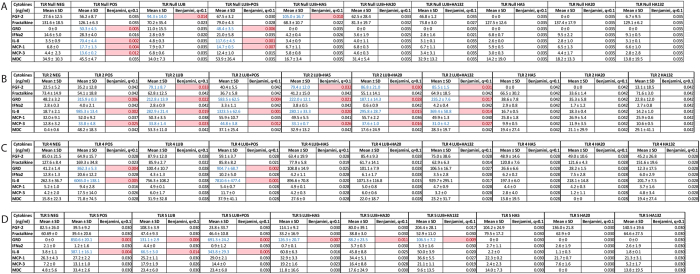
Lubricin and not HA regulates the inflammatory response through TLRs in HEK cells. Lubricin and/or HA was added to cells expressing specific TLRs and the supernatant was examined using Luminex Multiplex analysis. Results are shown as p values and significance (pink) is set to 0.05. The false discovery rate (*q*) was set at 0.1.

**Figure 3:**
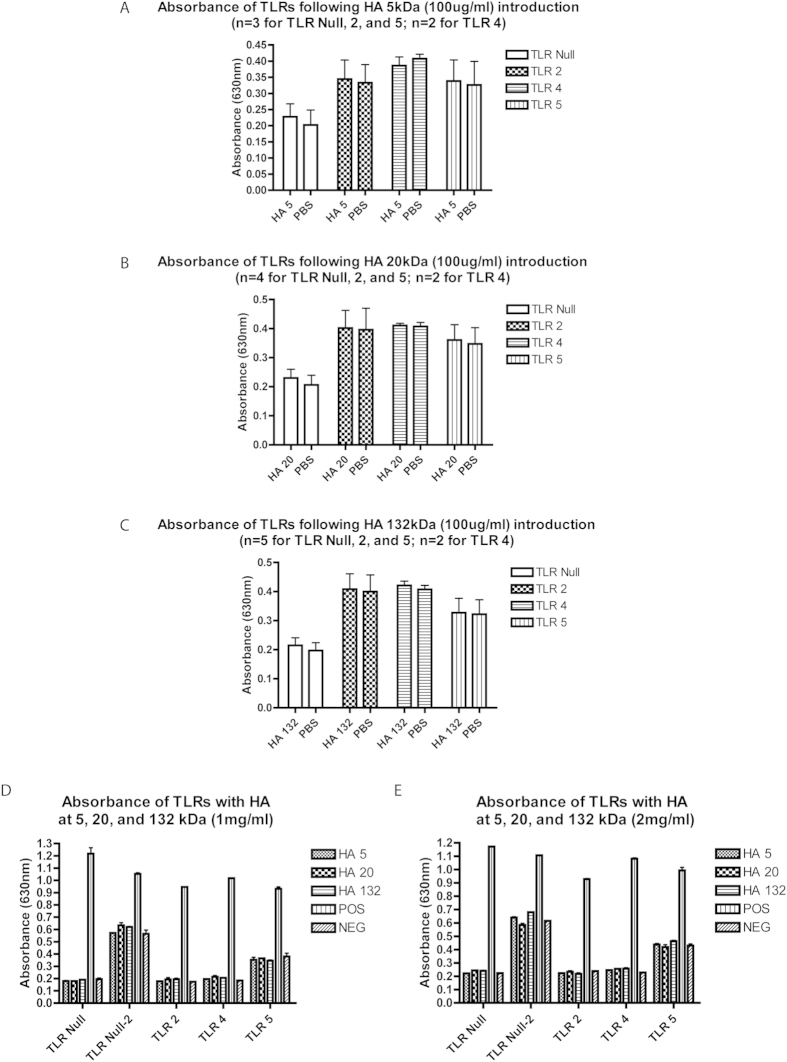
HA activation of NF-ĸB signaling through TLRs. The absorbance of TLR 2, 4, and 5 following the introduction of HA at MWs 5, 20 or 132 kDa. No effects on the TLRs were observed for the HAs regardless of fragment size.

Although a potential role for lubricin in regulating inflammation has been previously suggested as it has been demonstrated that polymorphonuclear leukocyte (PMNs) can be coated by lubricin, that subsequent exposure to TNF-α can lead to the ‘shedding’ of lubricin from these cells^38^, and that lubricin can bind to CD44 and regulate the activity of synovial fibroblasts^33^; the functional consequence of these mechanisms remains to be understood. Therefore, since TNF-α and CD44 are both active in TLR signalling, we performed a TLR- NF-ĸB screen with lubricin as a ligand. It was observed that lubricin was able to induce the translocation of NF-ĸB in HEK cells that over-expressed TLR 2, 4, 5 and 9 (Fig. 1). Since TLR 9 is an intracellular TLR, it was excluded from further examination in this study. It was next demonstrated that lubricin binds directly to, and activates NF-ĸB through TLR 2, 4 and 5 in a dose dependent manner (Fig. 1). Lubricin binding to TLR 2 and 4 demonstrated similar binding affinities of 3.3(±1.2)^×10-7^ M and 7.4( ± 2.0)^ × 10-7^ M respectively. The lubricin to TLR 5 binding interaction was stronger at 7.5(±1.5)^ × 10-8^M. After lubricin binding, the cytokine secretion profile of the HEK cells was altered (Fig. 2). Interestingly, and contrary to the published data^39^,^40^,^41^, low molecular weight HA did not result in activation of TLR 2, 4 or 5 in these cells and no NF-ĸB translocation was observed at either 100 ug, 1 mg or 2 mg per mL (Fig. 3). Furthermore, no differences in NF-ĸB activation (supplementary figure 1) or inflammatory cytokine expression (Fig. 2) were observed when HA and lubricin were combined, compared to lubricin alone. Additionally, no effects on TLR activation were observed when higher molecular weight HA were tested (supplementary figure 2). Multiple HA polymers with increasing molecular weight (5, 20, 132 and 780 kDa) were unable to activate NF-ĸB translocation in the presence of TLR 2, 4 or 5 and no change in cytokine secretion profile occurred in the presence of HA.

### Lubricin Conditionally Decreases NF-ĸB Translocation in the Presence of LPS and FLA

**Figure 4:**
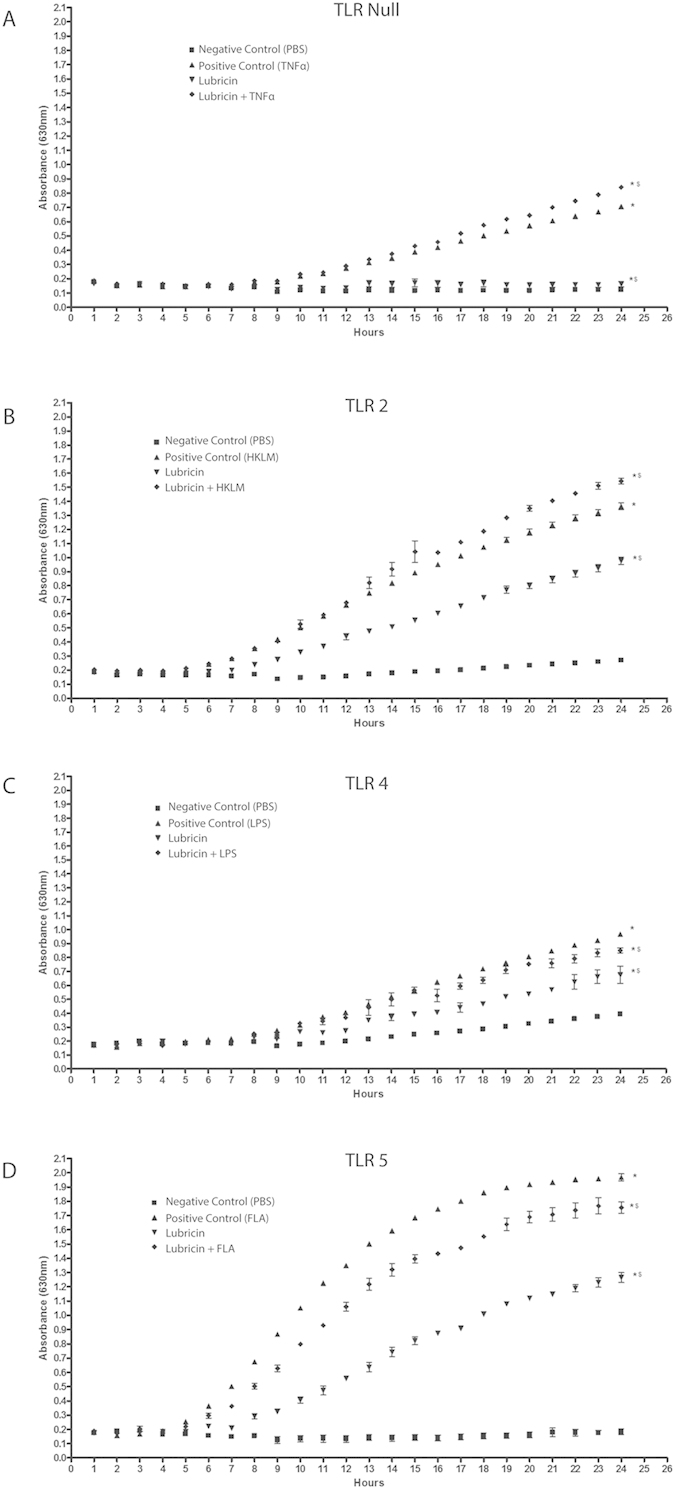
Lubricin regulates TLR activation triggered by TLR agonists. Lubricin does not induce NF-ĸB activation in the absence of TLRs (**A**), but does enhance TLR-2 activation in the presence of HKLM (**B**). However, in TLR-4 and -5 expressing cells, when lubricin was added alongside the corresponding agonist, a decrease in LPS (**C**) and FLA (**D**) mediated activation was observed.

While it was observed that lubricin enhanced the translocation of NF-ĸB in TLR-2 cells treated with HKLM, lubricin also decreased NF-ĸB translocation in the presence of LPS or FLA (Fig. 4). This effect was also observed in the inflammatory responses of the HEK cells (Fig. 2). Specifically, while, LPS increased the expression of GRO and IL-8 in TLR-4 positive cells; lubricin did not trigger these same responses (Fig. 2). Similar to the effect observed in the previous NF-ĸB translocation experiments with HA, HA when combined with the various TLR agonists had no detectable effect on the translocation of NF-ĸB (supplementary figure 3).

### Lubricin Decreases Expression of Cytokines from OA Fibroblasts

**Figure 5:**
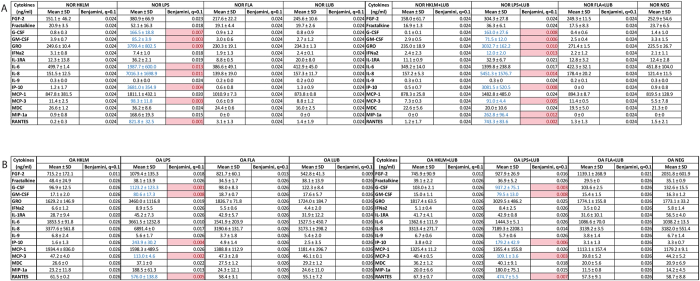
Lubricin–TLR interaction regulates cytokine expression in normal and OA synovial fibroblasts. In both normal (**A**) and OA (**B**) derived synovial fibroblasts the effect of lubricin treatment of cytokine expression is distinct from TLR agonists (HKLM, LPS, FLA), and in some cases when lubricin and an agonist are added together the response is different from each alone. Results are shown as p values and significance (pink) is set to 0.05. The false discovery rate (*q*) was set at 0.1.

Human synovial fibroblasts from normal and OA knee joints were examined to determine if lubricin was able to regulate the inflammatory response of these cells. To confirm previous findings^43^,^44^, flow cytometry was used to demonstrate that a subset of both normal and OA fibroblasts expressed TLR -2, -4 or -5 *in vitro* (representative data shown, supplementary figure 4), with a greater percentage of cells from the OA population expressing TLR-2 and -5 with a concurrent increase in expression of TLR- 2 and -5 per cell. No differences in TLR 4 expression in the population or per cell were observed between normal and OA fibroblasts (combined data from all 4 normal vs. all 4 OA cell lines, supplementary figure 4). This result was confirmed through examination of human synovial biopsies showing that TLR -2, -4 and -5 are expressed at a higher level by OA synovium compared to normal synovium *in vivo* (supplementary figure 5). After lubricin treatment of normal and OA synovial fibroblasts, no changes in the expression of inflammatory cytokines were observed (Fig. 5). We also observed that both normal and OA cells were not responsive to HKLM or FLA, but a number of cytokines and chemokines were up-regulated with LPS treatment (Fig. 5). Interestingly, we found that while lubricin did not modify cytokine expression on its own in normal cells, it did modify the expression of a number of proteins (IFNa2, IL-6, and MIP-1a) in the presence of LPS (Fig. 5), and this effect was not observed in OA cells. We also examined if lubricin triggered translocation of NF-ĸB in normal vs. OA synovial fibroblasts. In normal and OA synovial fibroblasts without lubricin treatment, very little NF-ĸB nuclear translocation was observed (supplementary figure 6). Interestingly, lubricin treatment of the normal synovial fibroblasts led to increased NF-ĸB translocation, but not to the same extent as HKLM, LPS or FLA (supplementary figure 6). OA synovial fibroblasts demonstrated a different response from normal cells, as NF-ĸB did not translocate in the presence of lubricin, HKLM, LPS or FLA (supplementary figure 6).

To examine if the response observed in human synovial fibroblasts was TLR dependent, TLR 2, 4 and 5 were knocked down in in the cells (supplementary figure 7) and the cytokine profile of the cells was examined (supplementary figure 7). It was observed that when TLR 2, 4 and 5 were knocked down in the same cells, that the cell exhibited a uniformly pro-inflammatory response to lubricin (supplementary figure 7).

### Lubricin Demonstrated Anti-inflammatory Effects *in vivo* and Decreased Pain in the Rat Following DMM

**Figure 6:**
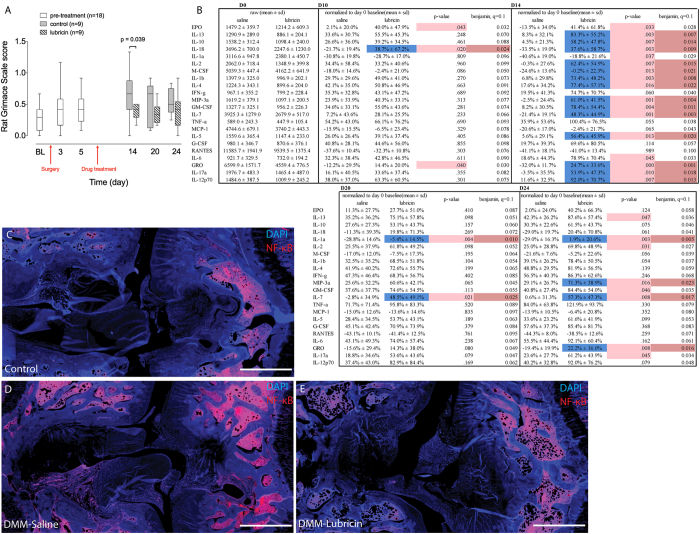
Analysis of inflammatory cytokines in serum and pain after DMM surgery and with Lubricin treatment. A single intra-articular administration of lubricin 7 days following joint injury (Destabilization of the Medial Meniscus) results in a significant and relevant decrease in RGS score (p = 0.04) at one week post injection. Data are displayed as Tukey box and whisker plots. (**A**). This corresponds with a decrease in a number of cytokines (**B**). NF-ĸB expression is limited in the marrow space in the sub-chondral bone in uninjured control rat knee joints (**C**). NF-ĸB is expressed throughout the injured rat knee joint (**D**). NF-ĸB expression is limited in the marrow space in the sub-chondral bone in lubricin injected injured rat knee joints (**E**). The false discovery rate (*q*) was set at 0.1.

Since synovial fibroblasts altered their inflammatory profile with exposure to lubricin, we next sought to determine if lubricin could regulate the inflammatory response in an animal model of OA. One week after DMM surgery in 18 rats, randomly selected animals received either an intra-articular injection of saline (n = 9), or lubricin (n = 9). Rats receiving lubricin showed maintenance of cartilage integrity after injury (supplementary figure 7), reduced osteophyte formation (supplementary figure 8), reduced synovial inflammation (supplementary figure 8) and reduced expression of cytokines (IL-13, IL-18, IL-10, IL-1α, IL-1β, IL-2, M-CSF, MIP-3a, GM-CSF, IL-4, IL-5, IL-7, GRO, IL-17a and IL-12p70) and also showed less pain as assessed with the Rat Grimace Scale (Fig. 6). Additionally, we were also able to observe an increase in lubricin staining at the cartilage surface of animals injected with lubricin compared to saline injected controls (supplementary figure 8). To investigate if a TLR-lubricin interaction was potentially the mechanism responsible for the deceased pain, we first determined that rats do express TLR- 2, -4 and -5 in multiple joint tissues (supplementary figure 9), and that under normal joint homeostatic conditions, limited NF-ĸB expression and virtually no translocation (except within the marrow space) was observed (Fig. 6, supplementary figure 10). However, after joint injury, NF-ĸB expression and translocation was observed in multiple joint tissues (Fig. 6, supplementary figure 11). When lubricin was injected into the rat joint after injury, NF-ĸB expression and translocation was inhibited to levels comparable to those in the uninjured joints (Fig. 6, supplementary figure 12). While this is not direct evidence that lubricin was interacting with the cells through TLR 2, 4 or 5, the decreases in NF-ĸB expression/translocation *in vivo*, do correlate with the decreased levels for a number of cytokines in the serum of the rats after lubricin injection (Fig. 6).

## Discussion

Taken together, these results suggest that: (**a**) lubricin is able to interact with TLR -2, -4 and -5 resulting in NF-κB activation, (**b**) this change in signalling leads to an alteration in cytokine/chemokine secretion, (**c**) this pathway may play a role in regulating TLR activation in the presence of classical and/or pro-inflammatory TLR ligands, and (**d**) this novel signalling interaction appears to be independent of HA. In this study, *in vitro* data have been provided that directly support these claims. However, we have also presented compelling *in vivo* data that suggests (although not directly) that lubricin also regulates the expression and localization of NF-ĸB, and this novel signalling pathway may play a role in joint pain.

While lubricin has not been previously implicated in TLR signalling, multiple DAMPS/Alarmins (high mobility group box protein 1, S100A8 and S100A9), degradation products of cartilage ECM and free fatty acids (i.e., DAMP’s) are all increased in OA joints^22^,^45^. The interaction of DAMP’s with the TLR receptor family in OA cartilage and synovium, including TLR-2, -4, -5 and others such as the receptor for advanced glycation end-products (RAGE)^46^ create a vicious cycle, as released cytokines can induce and release additional DAMPs^47^. Therefore, it has been hypothesized that the induction and amplification of inflammatory responses play a role in OA progression. Specifically, TLR-2 has been implicated in the pathogenesis of OA as it is upregulated in human OA synoviocytes and drives the expression of multiple inflammatory cytokines^24^. It has also been observed that Gc-globulin, α1 and α2-microglobulin can induce TLR4-dependent production of inflammatory cytokines and growth factors, including TNFα, IL-6, IL-1β and VEGF^48^.

Analyses of both human and rodent OA synovium have revealed increased levels of S100A8 and S100A9^49^ and these proteins are capable of inducing TLR-4 dependent cartilage catabolism via up-regulation of MMPs 1, 3, 9, and 13, as well as the pro-inflammatory cytokine IL-6 with concomitant inhibition of ECM protein expression^50^. Moreover, it has been observed that disease severity was reduced in S100A9-deficient animals in a collagenase-induced OA model^48^.

In our rat OA study, we found that a number of cytokines and chemokines downstream of NF-ĸB were up-regulated after injury and remained so after 4 weeks when the animals had developed OA (Fig. 6). While this result does not directly implicate the NF-ĸB pathway directly, it demonstrates that elements downstream of NF-ĸB are playing a role in the onset and progression of OA in a rodent model. We have also shown that lubricin administration to the joint after induction of OA helps maintain cartilage health (Fig. 3), leads to a decrease in pain in addition to lowered inflammation (Fig. 6).

As mentioned earlier, lubricin has never been shown to signal through TLRs, but HA has been reported to do so^24^. Lubricin and HA act synergistically in terms of lubrication^25^ and although HA does not ‘bind’ to lubricin, the two molecules ‘interact’ *in vivo* and *in vitro*. Therefore, it was critical to determine if the results we observed were a consequence of HA contamination of lubricin. To demonstrate this, we employed highly purified lubricin and HA (research grade), either alone or in combination, and screened these against cells overexpressing TLR-2, -4 or -5. In this experiment, we found that across various molecular weights HA alone was not able to activate TLR-2, -4, or -5 signalling through NF-κB (Fig. 3) and furthermore, no synergy was observed when HA was mixed with lubricin and presented to the cells (fig. 2, supplementary figure 1). This suggests that HA is not a direct ligand for TLR receptors (at least -2, -4 or -5) as recently suggested by Ebid *et al.*, 2014^42^, but serves as a co-factor, while lubricin may actually be the TLR ligand.

Taking these experiments one step further, we next attempted to uncover how lubricin might be signalling through TLR receptors using a competition assay between lubricin and common ligands for TLR2 (HKLM), -4 (LPS) or -5 (FLA). The TLR activation response between TLR ligand alone vs. lubricin alone was different and when TLR ligands were mixed with lubricin, the NF-κB response was different than either molecule alone (Fig. 4). Additionally, we were able to demonstrate that the cytokine/chemokine secretion profile of the cells was also different under each of these conditions (Fig. 2). Results from primary human synovial fibroblasts from normal and OA joints demonstrated similar results (Fig. 5, suggesting that lubricin is able to modify TLR signalling when the receptor is activated by something other than *in vivo* lubricin (e.g. TLR agonist). When TLR 2, 4 and 5 were knocked-down in normal human synovial fibroblasts, lubricin treatment initiated a strong pro-inflammatory response by the cells, suggesting that lubricin binding to TLR does keep the cell in a homeostatic or potentially anti-inflammatory state and that when these TLRs are disrupted or bound by other factors lubricin might act through additional signaling pathways that do not appear to be NF-ĸB dependent (based on the HEK data) to trigger the release of pro-inflammatory signals.

This study is not without its limitations. Although the trends in NF-ĸB activation and secretion profile of HEK vs. primary derived synovial fibroblasts were conserved, the levels and specific cytokines/chemokines were not. There are a number of reasons why this may be occurring; one might be the differences in signalling pathways between transformed and primary cells, specifically elements of the TLR activation cascade and/or downstream elements of the NF-ĸB pathway may be differentially regulated. It is also possible that the genetic modifications used to overexpress specific TLRs in the HEK cells may have altered the sensitively or specificity of the pathway downstream of the receptor. Specifically in the HEK cells, we cannot be certain which member of the downstream pathway is now the ‘limiting reagent’ as the receptors have been overexpressed. It is possible that by simply changing the ratio of receptor to intracellular signaling molecule (e.g. MYD88), the cell responds in a different way compared to a non-modified cell. However, these results clearly demonstrate that lubricin can bind to and activate TLR 2, 4 and 5 and that this activation can lead to alternations in the inflammatory response of these cells. Furthermore, the *in vivo* experiments do not demonstrate a direct link between lubricin, TLR and NF-ĸB. While transgenic mice deficient in a specific TLR could be used to study these relationships, the polyvalent binding of lubricin to multiple TLR receptors, will render this problematic.

Overall, the results presented strongly suggest that lubricin may be playing a larger role in joint homeostasis in addition to biolubrication, and that further studies are required to understand details of how such a novel lubricin–TLR signalling pathway is regulated under normal conditions and/or during the onset and progression of chronic joint diseases such as OA.

## Additional Information

**How to cite this article**: Iqbal, S.M. *et al.* Lubricin / Proteoglycan 4 binds to and regulates the activity of Toll-Like Receptors *In Vitro*. *Sci. Rep.* **6**, 18910; doi: 10.1038/srep18910 (2016).

## Supplementary Material

Supplementary Information
