# Supplementary material for: Lubricin/Proteoglycan 4 binds to and regulates the activity of Toll-Like Receptors In Vitro
Source: Sci Rep. 2016 Jan 11;6:18910. doi: 10.1038/srep18910 (PMC4707532; doi:10.1038/srep18910)
Supplement: Supplementary Information [file srep18910-s1.doc]

Lubricin / Proteoglycan 4 binds to and regulates the activity of Toll-Like Receptors *In Vitro*

**Authors:** S.M. Iqbal^1^, C. Leonard^1^, S. Regmi^2^, D. De Rantere^3^†, P. Tailor^1^, G. Ren^1^, H. Ishida^4^, CY. Hsu^1^, S. Abubacker^1^, D. SJ. Pang^3^, P. Salo^1^, H.J. Vogel^4^, D.A. Hart^1^, C.C. Waterhouse^5^, G.D Jay^6^, T.A. Schmidt^1,2,7^, R.J. Krawetz^1^*

**Affiliations:**

^1^ McCaig Institute, Cumming School of Medicine, University of Calgary, Calgary, Alberta, Canada.

^2^ Faculty of Kinesiology, University of Calgary, Calgary, Alberta, Canada.

^3^ Faculty of Veterinary Medicine, University of Calgary, Calgary, Alberta, Canada.

^4^ Faculty of Science, University of Calgary, Calgary, Alberta, Canada.

^5^ Snyder Institute, Cummings School of Medicine, University of Calgary, Calgary, Alberta, Canada.

^6^ Faculty of Medicine, Brown University, Providence, Rhode Island, United States.

^7^ Schulich School of Engineering, University of Calgary, Calgary, Alberta, Canada.

*Correspondence to: Dr. Roman Krawetz. Cummings School of Medicine. University of Calgary. HRIC 3AA14, 3330 Hospital Dr. NW. Calgary, Alberta, Canada. T2N 4N1. [rkrawetz@ucalgary.ca](mailto:rkrawetz@ucalgary.ca)

†Current Address: Western College of Veterinary Medicine, University of Saskatchewan.


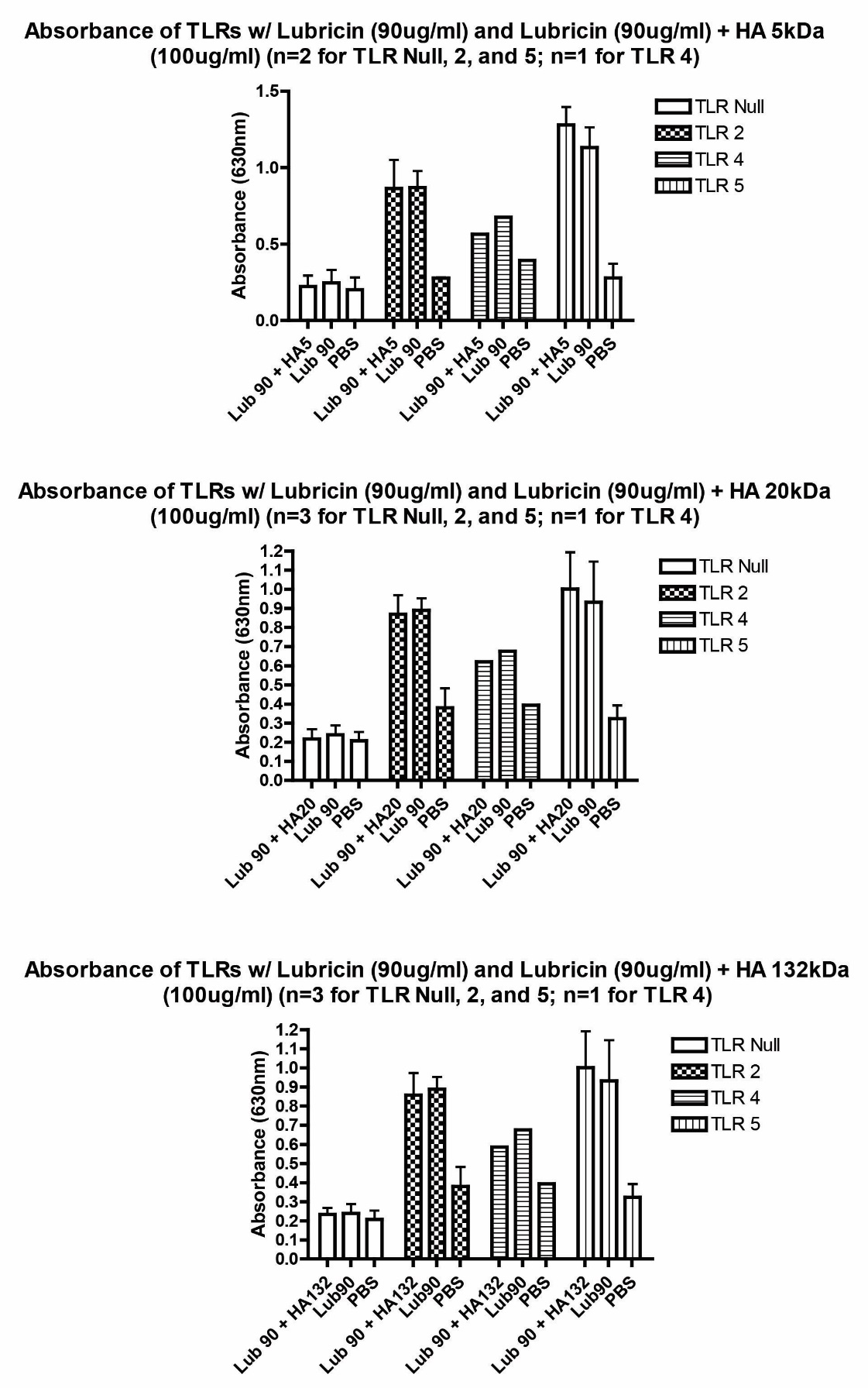


**Figure S1. NF-ĸB activation through TLR 2, 4 and 5 in the presence of lubricin and HA.** The absorbance for the different HA MWs were also tested in combination with lubricin (90ug/ml). No effects were observed for the HAs when present with lubricin irrespective of fragment size.


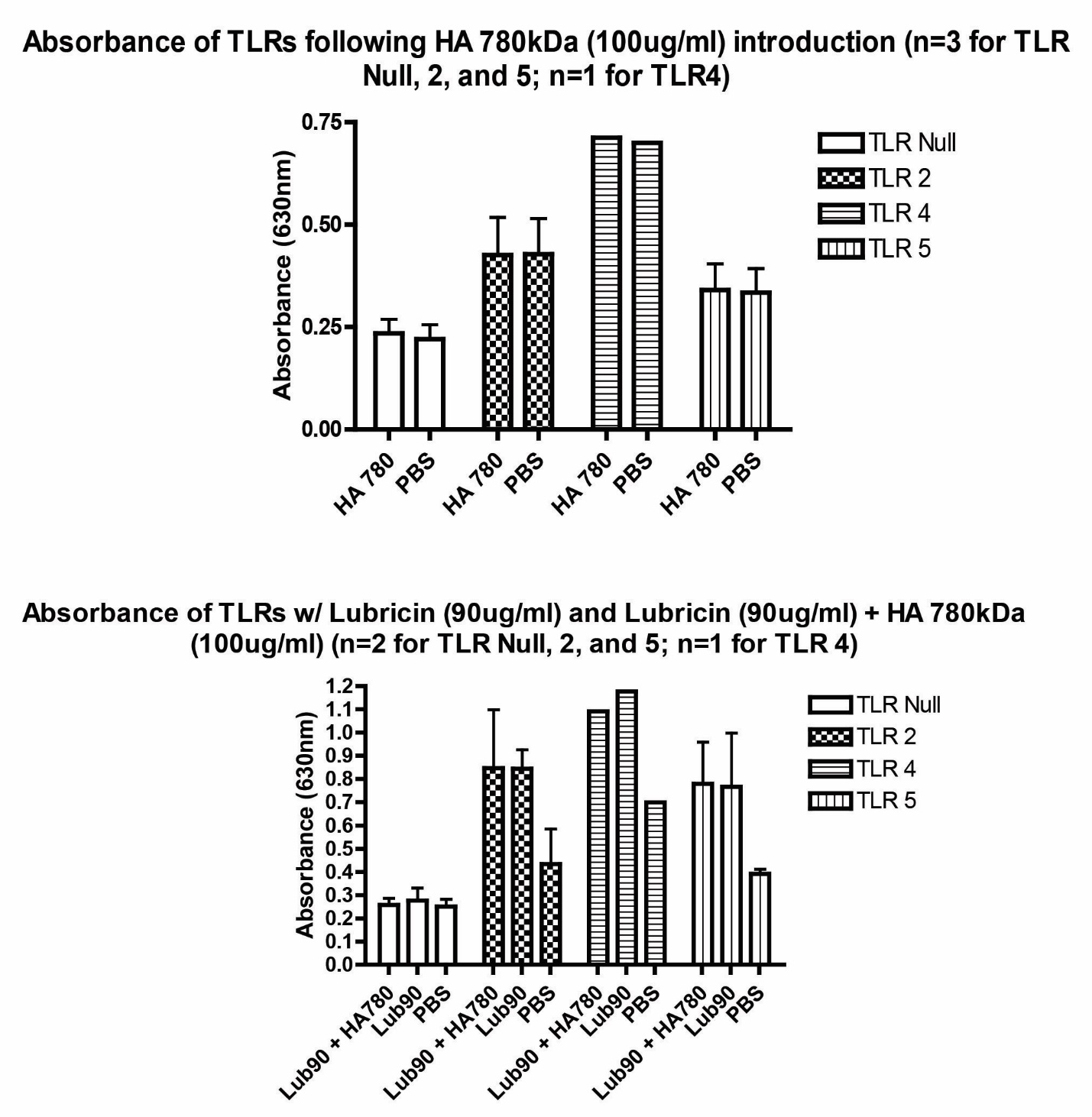


**Figure S2. NF-ĸB activation through TLR 2, 4 and 5 in the presence of lubricin and high molecular weight HA.** The absorbance for high MW HA (780kDa) was observed by itself or in combination with lubricin (90ug/ml) and no effects of HA was observed.


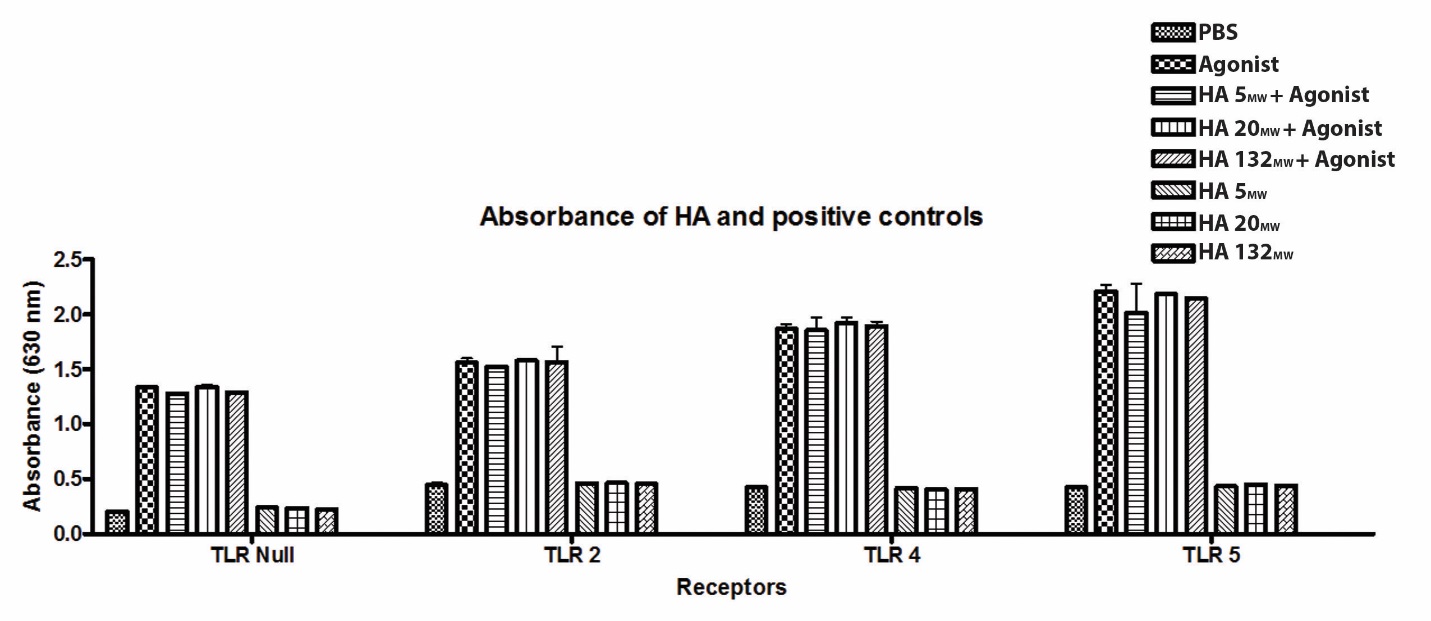


**Figure S3. NF-ĸB activation through TLR 2, 4 and 5 in the presence of HA and TLR ligands.** The absorbance of the different HA MWs were tested in the presence of the respective positive controls. No effects were observed for the HAs when present with the positive controls regardless of fragment size.


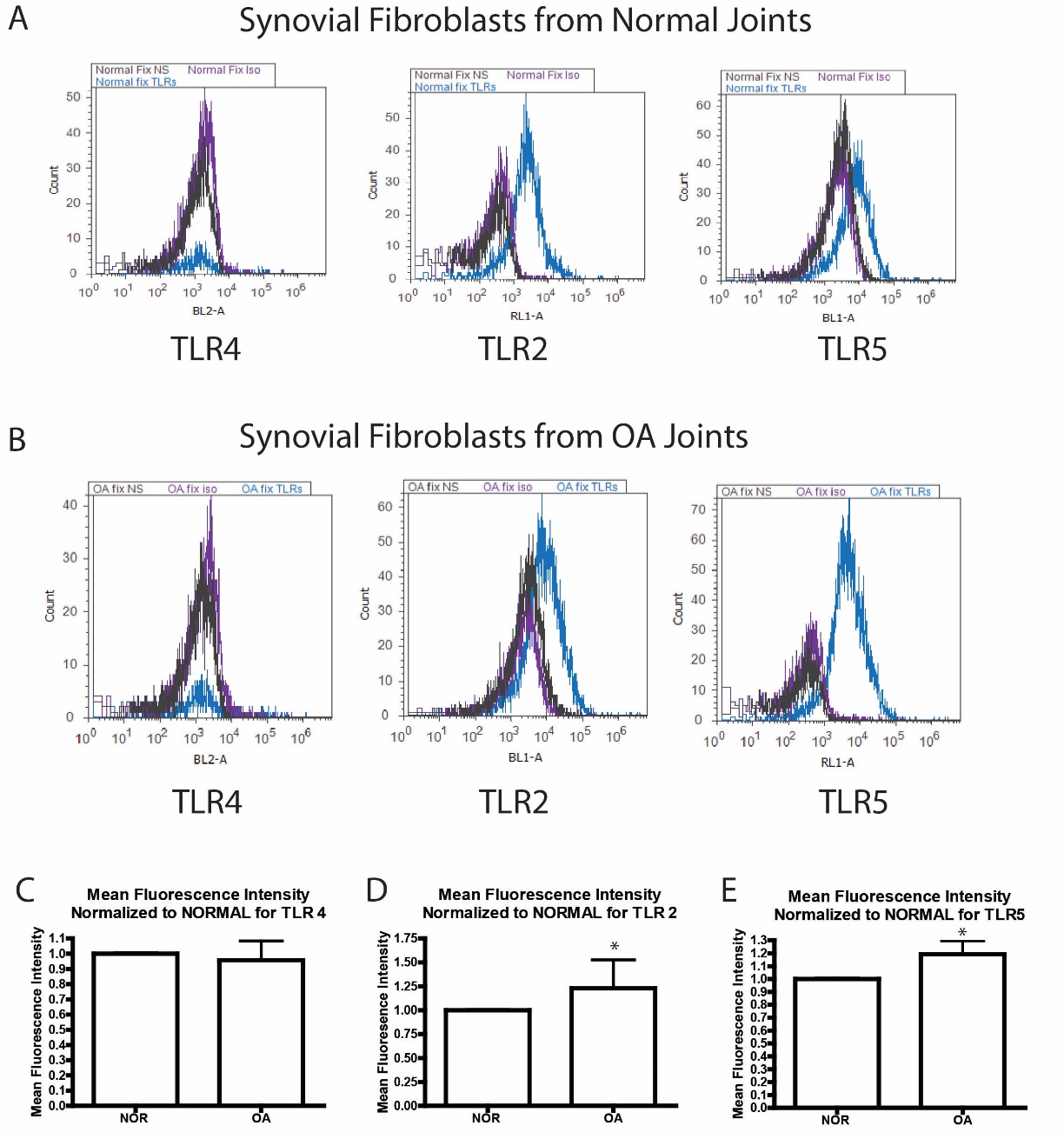


**Figure S4. Synovial fibroblasts from normal and OA joints express TLRs.** Synovial fibroblasts from normal (A) and OA (B) joints expressed TLR 2, and 5 with only moderate expression of TLR 4. Although no significant difference was observed in the mean expression per cell, there seemed to be a trend in greater expression of TLR 2 (D) and TLR 5 (E) in OA cells. *p=0.05.

**
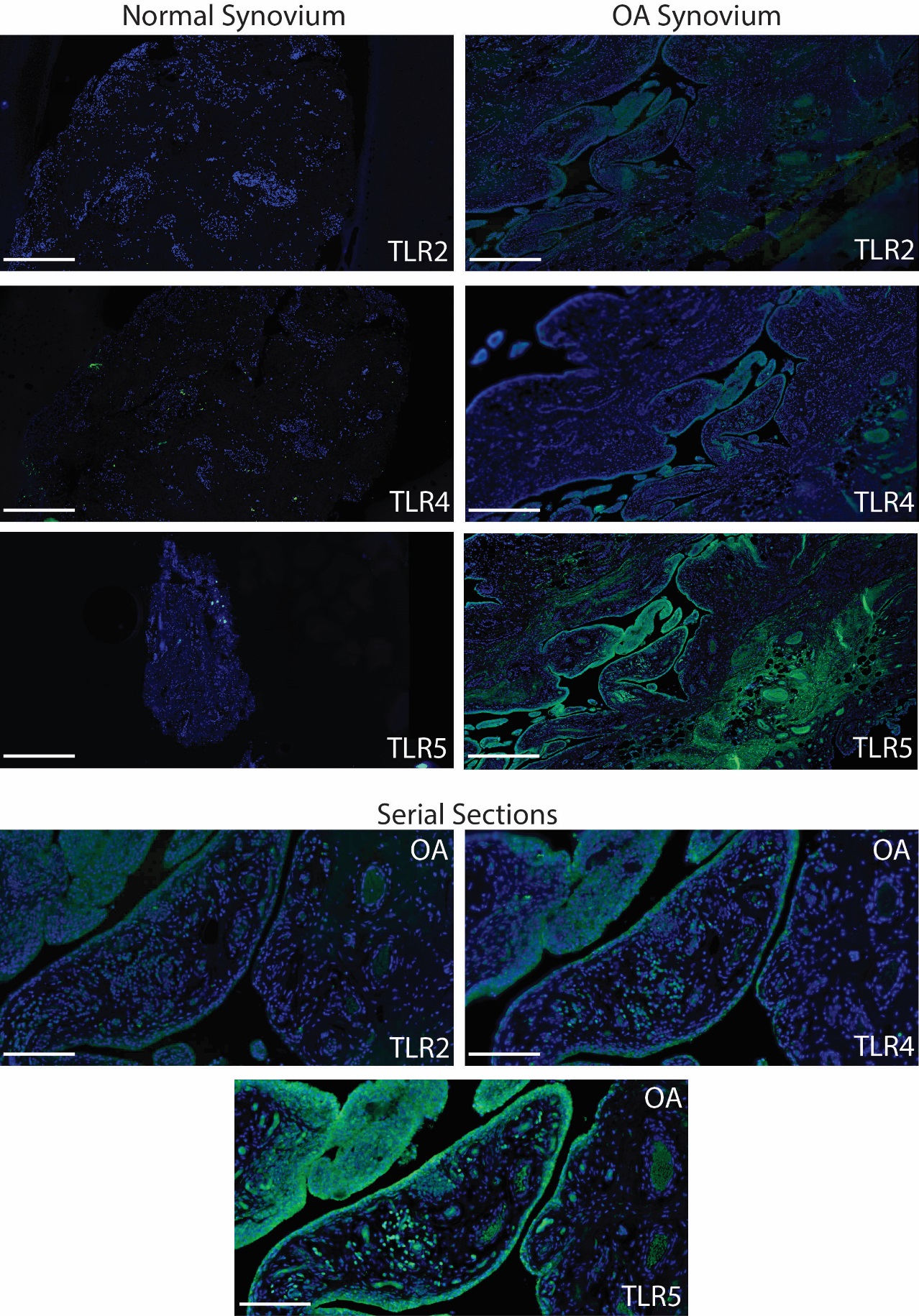
**

**Figure S5. TLR expression is present in human synovium.** Human synovial was collected from normal and OA joints and stained with TLR 2, 4 and 5. While little expression was detected in normal synovium, clear staining of all TLRs examined was observed in OA synovium.


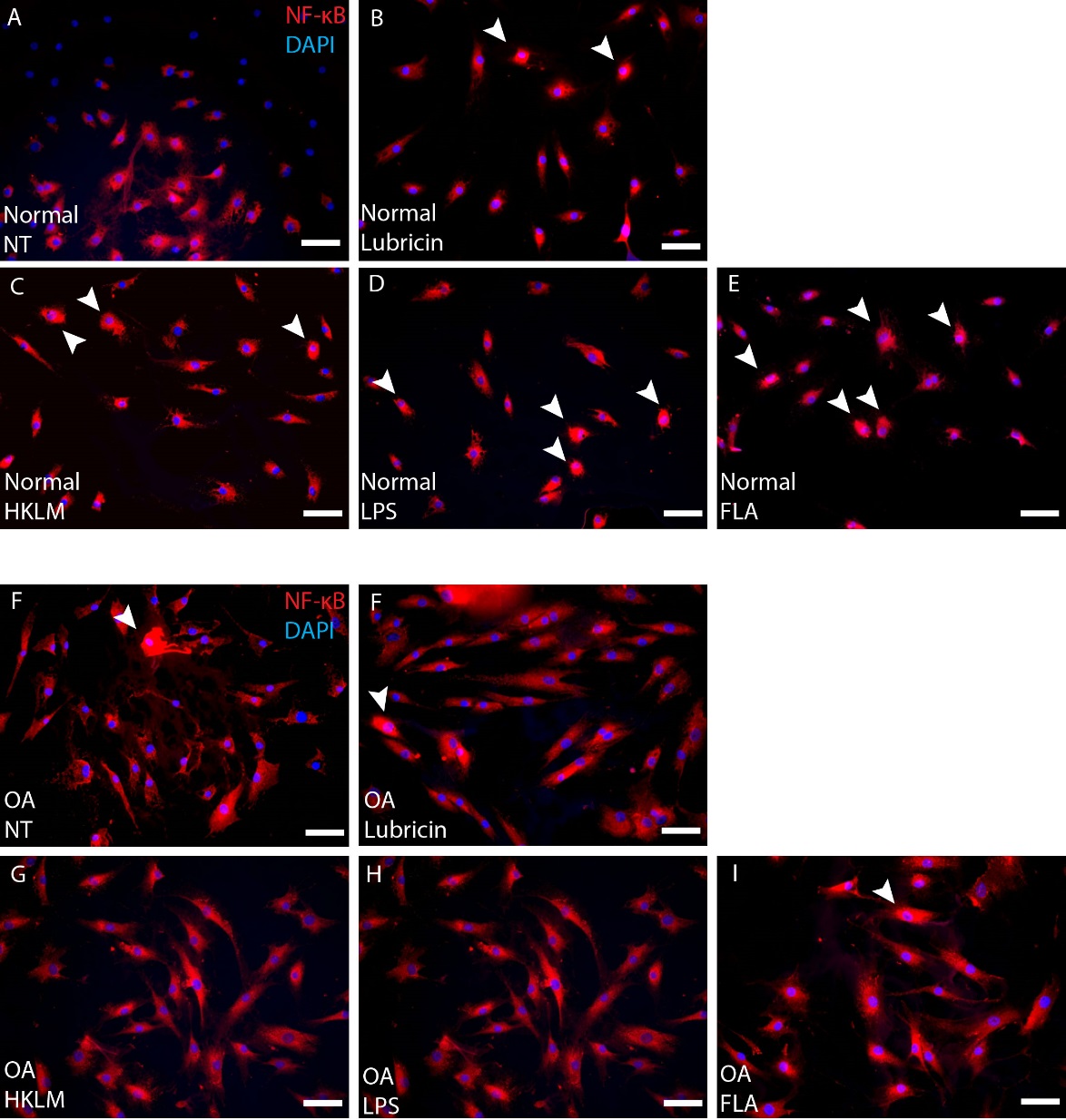


**Figure S6. There is a differential response in NF-ĸB nuclear translocation to lubricin and TLR agonists in normal and OA synovial fibroblasts.** Synovial fibroblasts from normal joints (A-E) demonstrate NF-ĸB translocation to lubricin (B), HKLM (C), LPS (D) and FLA (E). Synovial fibroblasts from OA joints, demonstrate little to no translocation in any treatment group. Scale bar equal 40um.


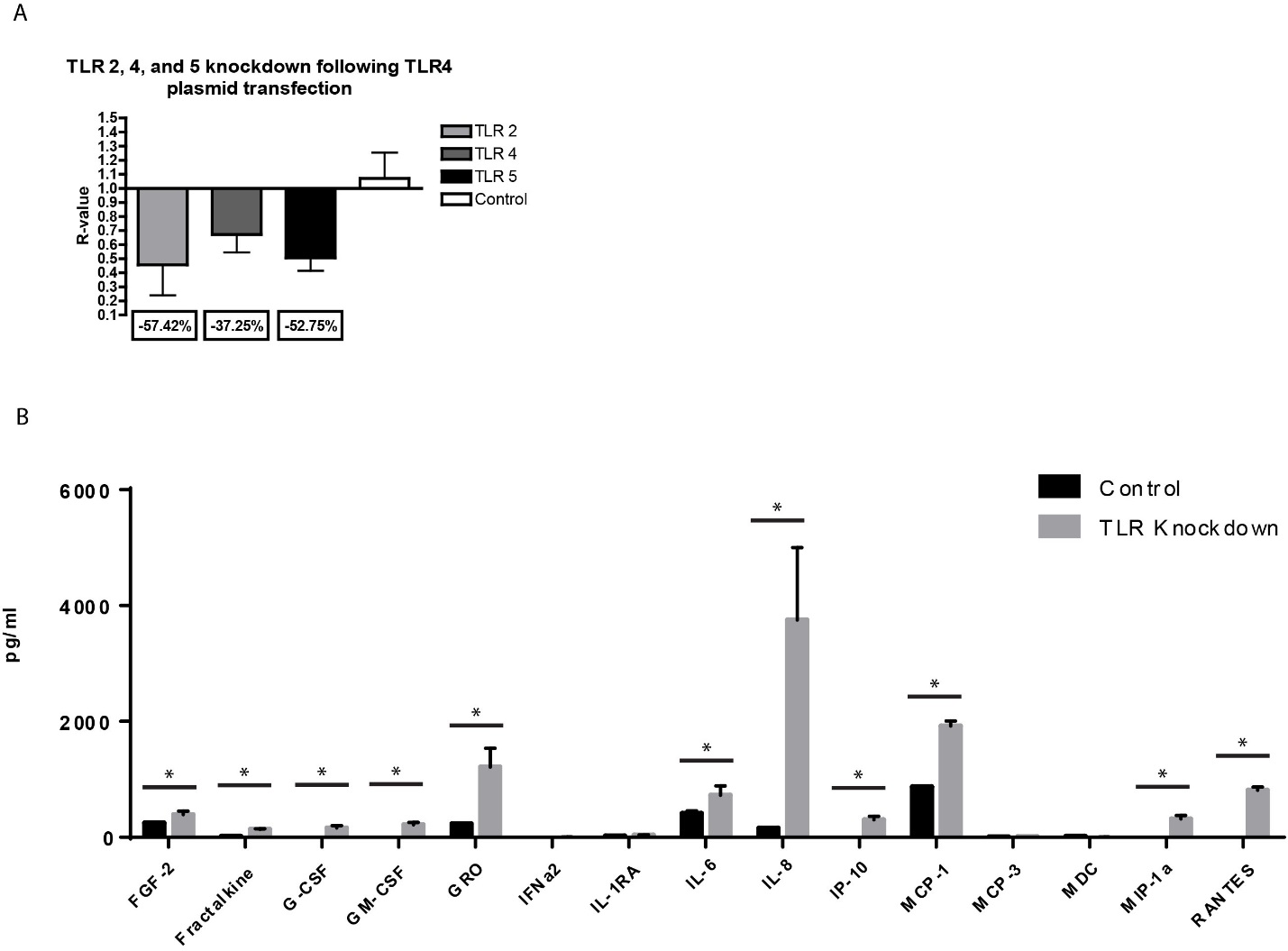


**Figure S7. Knock-down of TLR 2, 4 and 5 in normal human synovial fibroblasts.** TLR 2, 4 and 5 demonstrated approx. 40-50% knock-out at the mRNA level after all three sHRNA plasmids were co-transfected (A). The supernatant of the cells was examined and it was observed that FGF-2, Fractakline, G-CSF, GM-CSF, GRO, IL-6, IL-8, IP-10, MCP-1, M1P-1a and RANTES were all significantly increased in the TLR knockdown cells compared to the parental lines after exposure to lubricin. * The false discovery rate (*q*) was set at 0.1.


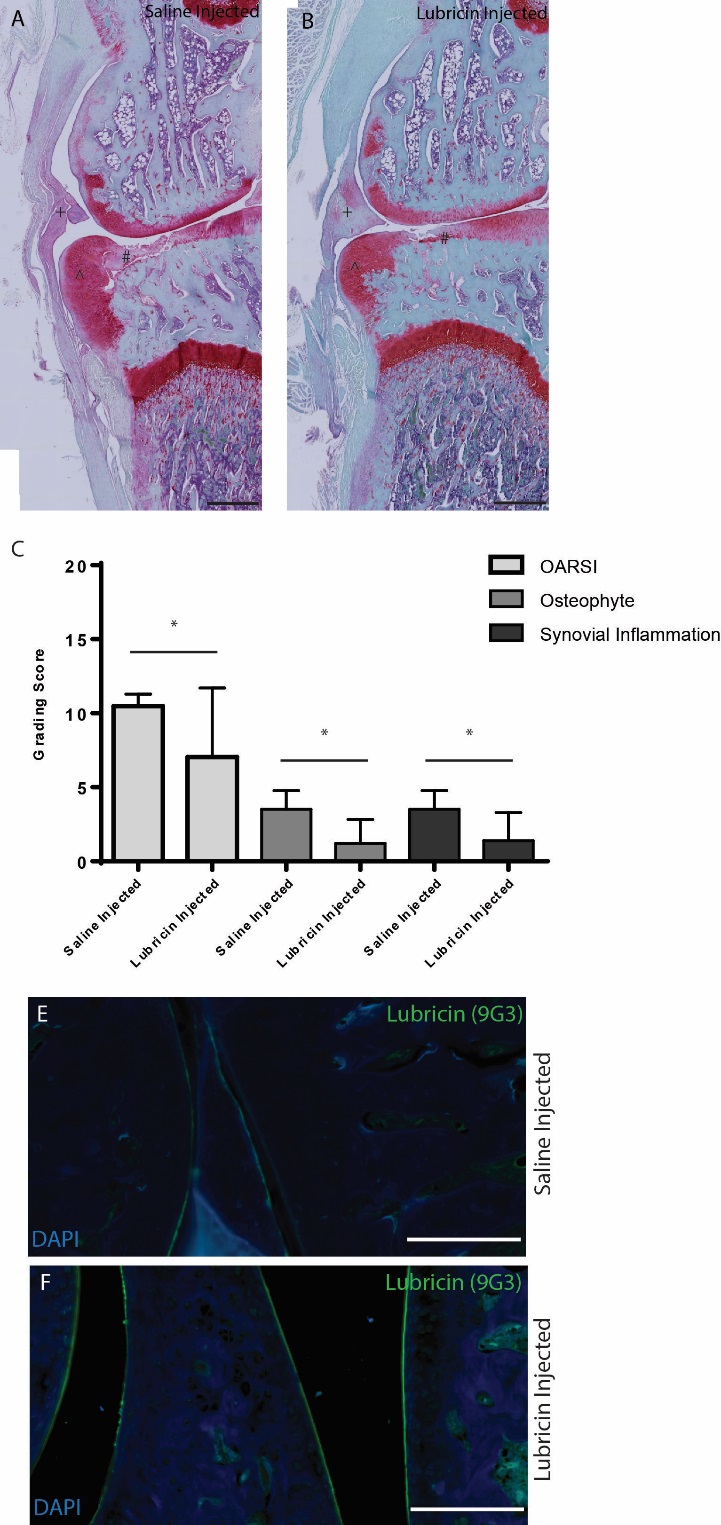


**Figure S8.** **Lubricin injection maintains cartilage health**. One week after DMM surgery either saline (A) or 200ug/kg lubricin (B) was injected into the joints of the rats. The rats injected with lubricin demonstrated significantly better cartilage histological (OARSI grading) outcomes, lower osteophyte scoring, and lower synovial inflammation grading (C). Lubricin expression was detected at the surface of the cartilage and meniscus in saline injected and lubricin injected animals. + = Synovitis, ^ = Osteophyte, # = Cartilage degeneration, p=0.05. Scale bar equals 200um.


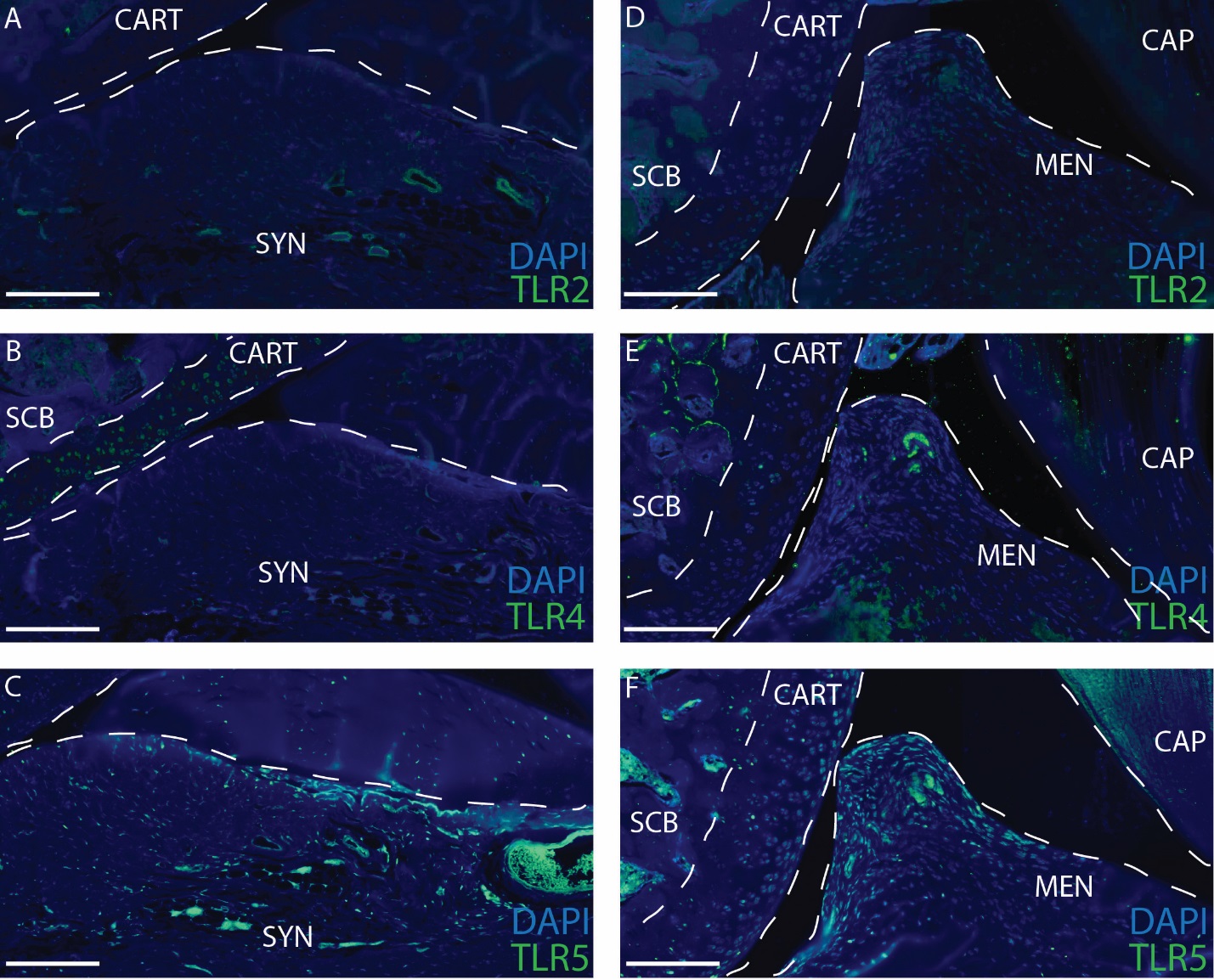


**Figure S9. TLR expression in the uninjured rat knee joint.** TLR 2, 4 and 5 staining was observed in both the synovium and cartilage/meniscus in control knee joints. Scale bar equals 150um.


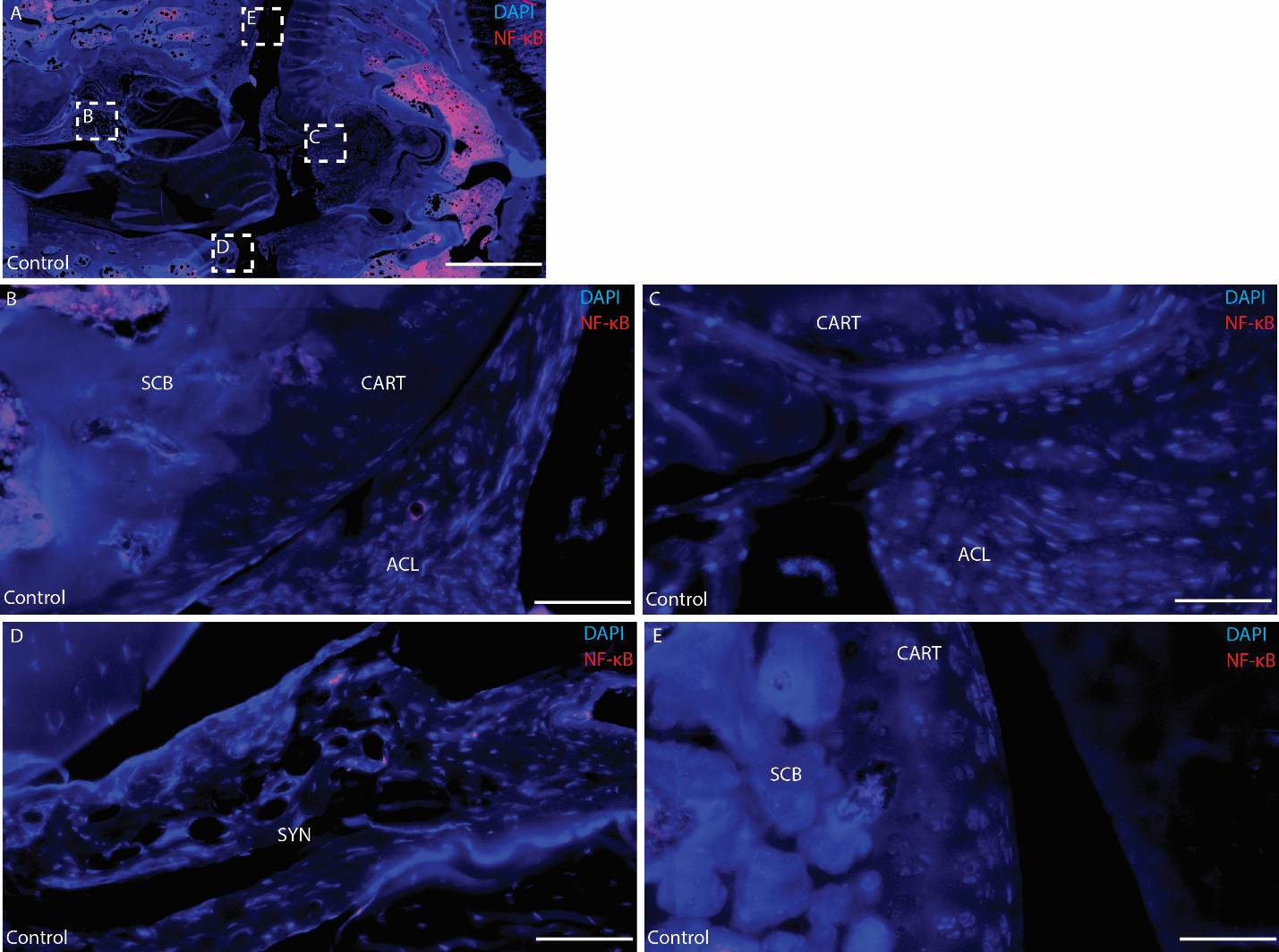


**Figure S10. NF-ĸB expression in normal rat joints.** NF-ĸB expression is limited in the marrow space in the sub-chondral bone in uninjured control rat knee joints (A). No NF-ĸB translocation is observed within any individual joint tissue. SCB = Sub-chondral bone, CART = Cartilage, ACL = Anterior Cruciate Ligament, SYN = Synovium.


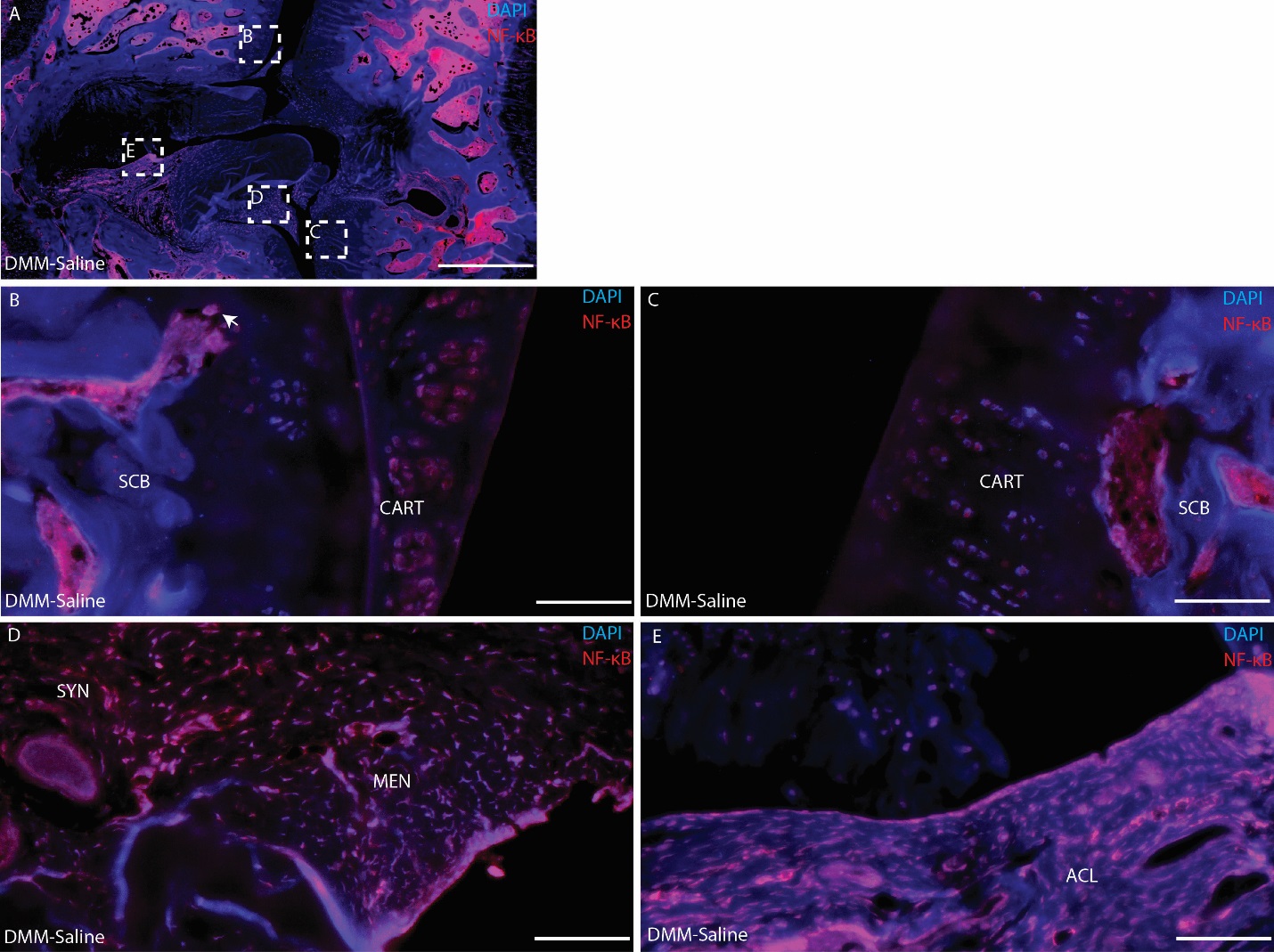


**Figure S11. NF-ĸB expression in OA rat joints.** NF-ĸB is expressed throughout the injured rat knee joint (A), and nuclear translocation can be observed is every joint tissue examined. SCB = Sub-chondral bone, CART = Cartilage, ACL = Anterior Cruciate Ligament, SYN = Synovium, MEN = Meniscus.


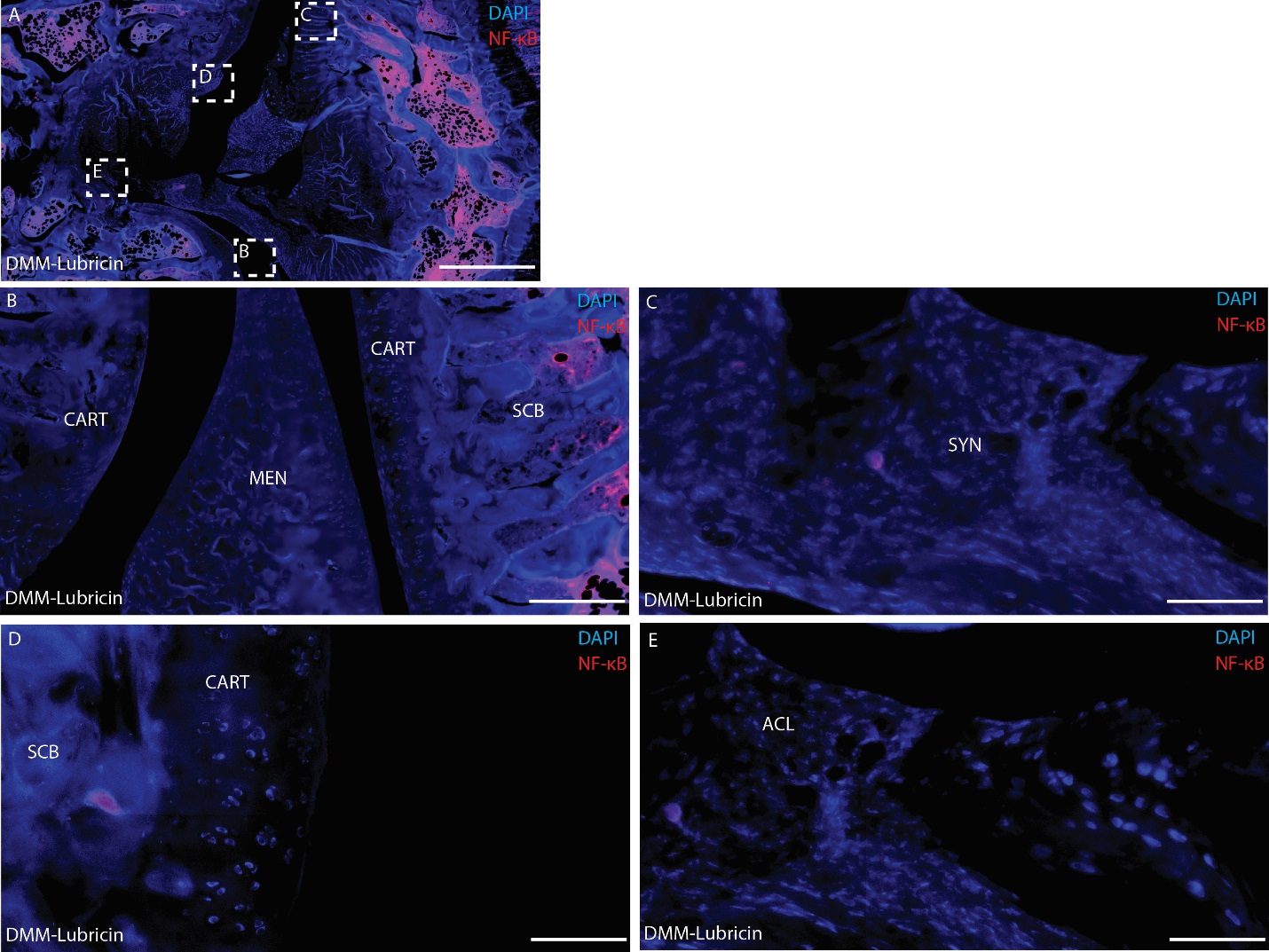


**Figure S12. NF-ĸB expression in OA rat joints injected with lubricin.** NF-ĸB expression is limited in the marrow space in the sub-chondral bone in lubricin injected injured rat knee joints (A). Very limited NF-ĸB translocation is observed within any individual joint tissue. SCB = Sub-chondral bone, CART = Cartilage, ACL = Anterior Cruciate Ligament, SYN = Synovium, MEN = Meniscus.
